# Effect of seawater temperature and pH on the sperm motility of the European eel

**DOI:** 10.1007/s10695-024-01311-y

**Published:** 2024-02-07

**Authors:** Malbelys P. Sanchez, Thales S. França, Wendy A. González-López, Marina Morini, Juan F. Asturiano, Luz Pérez

**Affiliations:** Grupo de Acuicultura y Biodiversidad, Instituto de Ciencia y Tecnología Animal, Universitat Politècnica de València, Valencia, Spain

**Keywords:** *Anguilla anguilla*, Global warming, Acidification, Sperm quality

## Abstract

The current climate change situation could bring critical effects for marine species, especially those already considered endangered. Although some species can adapt fast to the environmental changes, it is necessary to get into the worst scenario and develop tools to anticipatedly assess the physiological effects of such environmental change. With this purpose, our study aims to determine the effect of a range of seawater temperatures and pHs on sperm motility in the European eel (*Anguilla anguilla*). Low seawater pH (6.5–7.4) decreased the eel sperm motility in comparison to the control (pH = 8.2). We also studied the combined effect of the pH of the artificial seminal plasma (the plasma where the sperm cells are suspended) with the pH of Artificial Sea Water (ASW, pH 7.8 or and 8.2). We did not find statistical differences in sperm motility and kinetic parameters caused by the artificial seminal plasma pH. However, seawater pH induced significantly higher values of total sperm motility, and the percentage of fast spermatozoa with a pH of 8.2 in comparison with a pH of 7.8. In contrast, the seawater temperature did not affect sperm motility parameters or sperm longevity. To study the effect of the interaction between seawater temperature and pH on sperm motility, two temperatures: 4 and 24 °C, and two pHs 7.8 and 8.2, were tested. There were significant differences between temperature and pH in several kinetic parameters and, in general, the lowest values were observed in the samples activated at low temperature and low pH (4 °C, pH 7.8). This work suggest that eel sperm motility and kinetics will not be affected by the expected changes in pH or temperature due to the climate change.

## Introduction

Climate change is inducing changes to the environmental conditions, including ocean warming, acidification, and deoxygenation. Marine fish would be probably affected, as it is known that at least temperature is one of the environmental factors affecting fish reproduction, both as a proximate factor (affecting the starting of the reproductive events) as well as a ultimate factor (affecting the final reproductive events, ovulation, and spermiation). Indeed, most fish species show external fertilization: their spermatozoa and eggs are delivered in the external environment (marine or freshwater), which represent both a drastic environmental change and a source of chemical signals controlling the motility function (Cosson 2019). When the spermatozoa are released into the surrounding water, they experience an osmotic shock, which could be either hypoosmotic (in freshwater spawners) or hyperosmotic (in seawater spawners) (Pérez 2020) and such an osmotic shock induces the initiation of sperm motility. Sperm motility is a key requirement to assess the quality and fertilizing capacity of the sperm (Billard et al. 1995).

Changes in the external medium can regulate sperm motility by interaction with factors such as temperature, pH, and several others. Because motility in fish spermatozoa is triggered externally, it highly depends on the environment where reproduction occurs (Dzyuba and Cosson 2014); therefore, we can expect the spermatozoa to be strongly influenced by the evironmental temperature and pH (Lahnsteiner and Caberlotto 2012).

Knowledge of the physiological mechanisms by which high water temperature disrupts fish reproduction could contribute to understanding potential alterations of captive fish breeding, and to assist in their prediction (Merino et al. 2023). Increased water temperature can generate negative effects on sperm function, altering the motility time and sperm velocity, increasing the rate of cell metabolism, and causing a mismatch of energy resources. These effects could promote changes in the movement of the sperm, jeopardizing their quality and fertilizing capacity (Cejko et al. 2016; Fenkes et al. 2017).

pH is an important environmental factor in seawater chemistry and in eel sperm physiology (Pérez et al. 2020), and it is known that climate change is decreasing the pH of marine waters. It is estimated that current mean seawater average pH is around 8.1, but it could decrease to values of 7.8 by the end of the century (Coley et al. 2022). In those aquatic species showing external fertilization, the pH of the activating medium to which they release spermatozoa also influences the initiation of sperm motility (Alavi and Cosson 2005). In several fish species, such as sturgeons (Gallis et al. 1991), pufferfish (Takai and Morisawa 1995), or Japanese eel (Tanaka et al. 2004), it has been proven that alkaline waters (pH 7.4–9.0) favor the activation of motility sperm, while acidic waters (pH 6.0–7.2) reduce or inhibit it.

The European eel has been classified on the Red List of the International Union for Conservation of Nature (IUCN) as “critically endangered” and therefore, may be one of the species greatly affected by climate change. However, it is not known which temperature and pH values could be critical and might endanger the reproduction of the European eel. This is a species with a peculiar life cycle in which pubertal individuals undertake, apparently in 6–7 months, a transatlantic migration to the spawning areas in the Sargasso Sea (Tesch 1978), during which they face important environmental changes (salinity and temperature) that modulate the endocrine control of their sexual maturation (Tesch 1978; Mazzeo et al. 2016). It is necessary to develop tools to assess the actual physiological effects of anticipated environmental changes and use them to anticipate and mitigate their potentially harmful effects. With these purposes, our study aims to determine the effect of environmental temperature and pH on the motility performance of the European eel sperm.

## Material and methods

### Fish maintenance and hormonal treatment

A total of 135 male eels (mean body weight 105.7 ± 4.2 g) in three batches of 45 males (2021, 2022) were transported to our facilities at the Universitat Politecnica de Valencia (Spain) from the local eel farm Valenciana de Acuicultura, S.A. (Puzol, Valencia). Each fish batch was distributed in three 96-L aquaria (approximately 15 male eels per aquarium) equipped with separate recirculation systems, thermostats, and coolers, and covered with black panels to reduce light intensity and fish stress. The animals were gradually acclimatized to seawater (salinity 37.0 ± 0.3 g/L) over the course of 1 week, and were then maintained in seawater at 20 °C until the end of the experiment.

Once the fish were in seawater, the hormonal treatment with recombinant human chorionic gonadotropin (hCGrec; Ovitrelle, Merck S.L., Madrid) was initiated as described in previous studies (Gallego et al. 2013; Pérez et al. 2020). Once a week, the animals were anesthetized with benzocaine (60 ppm) and weighed before receiving an intraperitoneal injection of hCGrec diluted in NaCl 0.9%, at a dose of 1.5 IU/g fish.

The fish were fasted throughout the experiment and handled in accordance with the European Union regulations concerning the protection of animals used for scientific purposes (Dir 2010/63/UE) and with the recommendations provided by the Guide for the Care and Use of Laboratory Animals of the Spanish Royal Decree 53/2013 regarding the protection of animals used for scientific purposes (BOE 2013). The Experimental Animal Ethics Committee from the Universitat Politecnica de Valencia approved the applied protocols, and final permission (2023-VSC-PEA-0039) was granted by the local government (Generalitat Valenciana).

### Sperm collection and sampling

Sperm samples were collected once a week, from the 6th week of hormonal treatment until weeks 18 (batch 1), 24 (batch 2), and 20 (batch 3). The samples were collected 24 h after the administration of the hormone to obtain maximum sperm quality (Pérez et al. 2000). The sperm was collected in 15-ml Falcon tubes by applying gentle abdominal pressure, following fish anesthetization (benzocaine, 60 ppm). The genital area was previously cleaned with distilled water, and dried, in order to avoid sample contamination by faeces, urine and seawater. The sperm samples were kept refrigerated (4 °C) until the motility analyses were performed within the first hour after collection.

### Sperm motility evaluation

The standard sperm diluent used in this work was P1, an artificial seminal plasma isosmotic and isoionic with the European eel seminal plasma (in mM: NaCl 125, NaHCO_3_ 20, MgCl_2_ 2.5, CaCl_2_ 1, KCl 30; osmolality 325 mOsm/kg and pH adjusted to 8.5 by using solutions of NaOH and/or HCl 1 M, 0.1 M, 0.01 M) (Asturiano et al. 2004; Peñaranda et al. 2010). Sperm motility activation was undertaken as described by Gallego et al. (2013), by mixing 1 μl of diluted sperm (dilution 1/25 in P1) with 4 μl of artificial seawater (ASW; Aqua Medic Meersalz, 37 g/l, with 2% BSA (w/v), pH adjusted to 8.2). The mixture was prepared in a SpermTrack-10® chamber, with a depth of 10 μm (Proiser R + D, S.L.; Paterna, Spain) and observed using a Nikon Eclipse 80i microscope, with a 10 × lens (Nikon phase contrast 10—0.25, Ph1 BM WD 7.0). Motility was recorded 15 s after mixing the sperm with ASW, using a high-sensitivity HAS-220 video camera (using a frame rate of 60 fps) and the ISAS software (Proiser R + D, S.L.; Paterna, Spain), a computer-assisted sperm analysis (CASA-Mot) system. The sperm motility of each sample at each pH was measured in triplicate (3 activations per sample), with two videos captured for each activation. Both the sperm and the ASW were maintained at 4 °C in a water bath until the sperm motility evaluation. Only the best samples (> 60% total motility) were selected for the experiments. The sperm motility parameter considered in these studies were total motility or percentage of motile cells (MOT, %), and spermatozoa were considered immotile if their VCL was < 10 m/s. Other kinetic parameters were also explored: progressive motility (MP, %), defined as the percentage of spermatozoa which swim forward in an essentially straight line; the percentage of fast spermatozoa (FA; showing an average path velocity, VAP > 100 m/s); curvilinear velocity (VCL, µm/s), defined as the time/average velocity of a sperm head along its actual curvilinear trajectory; straight line velocity (VSL, µm/s), defined as the time/average velocity of a sperm head along the straight line between its first detected position and its last position; VAP (µm/s), defined as the time/average of a sperm head along its spatial average trajectory; straightness (STR, %), defined as the linearity of the spatial average path (VSL/VAP); ALH, amplitude of the lateral movement of the sperm head, and cross beating frequency (BCF; beats/s), defined as the average rate at which the curvilinear sperm trajectory crosses its average path trajectory.

### Experiments

#### Experiment 1. Effect of seawater pH on sperm motility and longevity of the sperm

This experiment was performed in three sessions, with 25 sperm samples from 25 fish in total. Sperm samples were first diluted in P1 as has been described previously. The pH of artificial seawater (ASW) was adjusted to the next pHs (± 0.02): 6.5, 7.2, 7.4, 7.6, 7.8, 8.0, 8.2, and 9.5. It is considered that a pH of around 8.1 is the standard pH of natural seawater, and our aim was to test the effect of lower pHs such as those that might occur due to climate change.

As there were no statistical differences in the sperm motility of the samples activated with ASW at pH of 7.6, 7.8, 8.0, and 8.2, we wanted to know if there were differences in the total time of motility (or longevity) between those pHs. We activated the sperm samples with ASW at pHs 7.6, 7.8, 8.0, and 8.2 and registered the total time of motility with a timer, until the spermatozoa moved less than 10% (checked by capturing videos with the CASA system every 30 s until less than 10% of spermatozoa were motile). This test was performed in two sessions with a total number of 14 samples (initial motility > 60%).

#### Experiment 2. The combined effect of seawater pH and diluent pH on sperm motility

To evaluate the possible interaction between the diluent pH and the seawater pH, we assayed the dilution of sperm in diluent P1 adjusted to 3 pHs: 8.5 (considered as the control), 8.0, and 7.5. The dilution was 1:25 (v/v). Samples (*n* = 16) were incubated at the different pHs of the extender for 1 h at 4 °C. Samples were evaluated later activating them with ASW at two different pHs: 7.8 and 8.2. The pH value of 7.8 was chosen since it is the seawater pH value expected for the year 2100 as a result of the climate change (Hartin et al. 2016).

#### Experiment 3. Effect of the seawater and extender temperature on sperm motility and kinetic parameters

This experiment was performed in a single session, with 10 sperm samples with good motility (> 60%). Each sample was diluted 1:25 in P1 and divided into two subsamples: one was maintained at 4 °C, and the other maintained at room temperature, approximately 23 °C. Samples were incubated at those temperatures for 1 h. Activation of of the sample maintained at 4 °C was undertaken with ASW also maintained at 4 °C, while the sample maintained at 23 °C was activated with ASW at that temperature. Motility and kinetic parameters were then measured.

#### Experiment 4. Effect of the seawater temperature on sperm longevity

To evaluate the effect of the seawater temperature on sperm longevity, this parameter was measured as described above. We selected 10 sperm samples for the analyses. Each sample was diluted 1:25 in diluent P1 (pH 8.5). Diluted samples were incubated at 4 or 23 °C for 1 h, and later activated using ASW at the same temperatures.

#### Experiment 5. The combined effect of pH and seawater temperature on sperm motility

In this experiment, sperm samples were selected and two ASW and diluent temperatures were tested: 4 and 24 °C (RT), as well as two pH values in the seawater: 7.8 and 8.2, resulting in four experimental combinations. Then, aliquots of ASW at 7.8 and 8.2, as well as the diluted samples, were maintained either at 24 °C or in the refrigerator at 4 °C for 1 h. This test was performed with a total number of 12 samples in three sessions. The sperm motility was evaluated, as previously described, with a CASA-mot system.

### Statistical analyses

Each variable was first analyzed to check its normality using the asymmetry and curtosis coefficients as reference parameters. For not normally distributed populations, Kruskal–Wallis one-way ANOVA on ranks and Mann–Whitney *U*-test were used.

When the experiment had two variables, a two-way ANOVA was performed first, and according to the results, a single ANOVA was then performed to evaluate the differences between the experimental treatments. The experiments with a single variable were analyzed by a one-way ANOVA. The means comparison was undertaken with the Duncan multiple range test. For all the tests, the differences were considered significant when *p*-value < 0.05. All the statistical procedures were performed with the software Statgraphics Plus® 5.1 (Statistical Graphics Corp., Rockville, MO, USA) and GraphPad Prism 9.3.0 (GraphPad Software, San Diego, California USA, www.graphpad.com). Results are presented as the mean ± standard error.

## Results

### Experiment 1. Effect of seawater pH on sperm motility and longevity in eel sperm

**Fig. 1:**
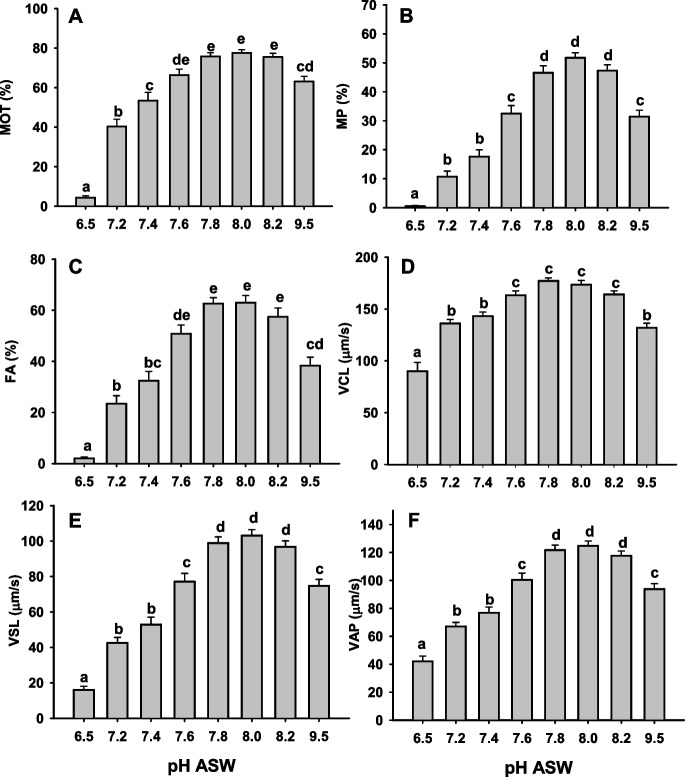
Effect of artificial seawater pH in the total motility (MOT), progressive motility (MP), percentage of fast spermatozoa (FA), curvilinear velocity (VCL), straightline velocity (VSL), and average path velocity (VAP). Values are represented as means ± standard error. *n* = 23. Different letters indicate significant differences between means (*p* < 0.05)

A trial was performed to test if a specific seawater pH generated any effect on the eel sperm motility in comparison to the control (pH = 8.2). Figure 1 shows how kinetic sperm values were higher in the pH range of 7.8–8.2 of ASW. When ASW became more acidic, kinetic values, including total motility (MOT) decreased. Also, at a high pH (9.5), there were lower MOT, MP, FA, VCL, VSL, and VAP values than those found with the pH 7.8–8.2. Thus, when ASW pH is between 7.8 and 8.2, there is a higher percentage of motile spermatozoa (MOT), a higher percentage of fast spermatozoa (FA), which move with higher velocities (VCL, VSL, VAP), and have a higher displacement in the water (progressive motility, MP).

**Fig. 2:**
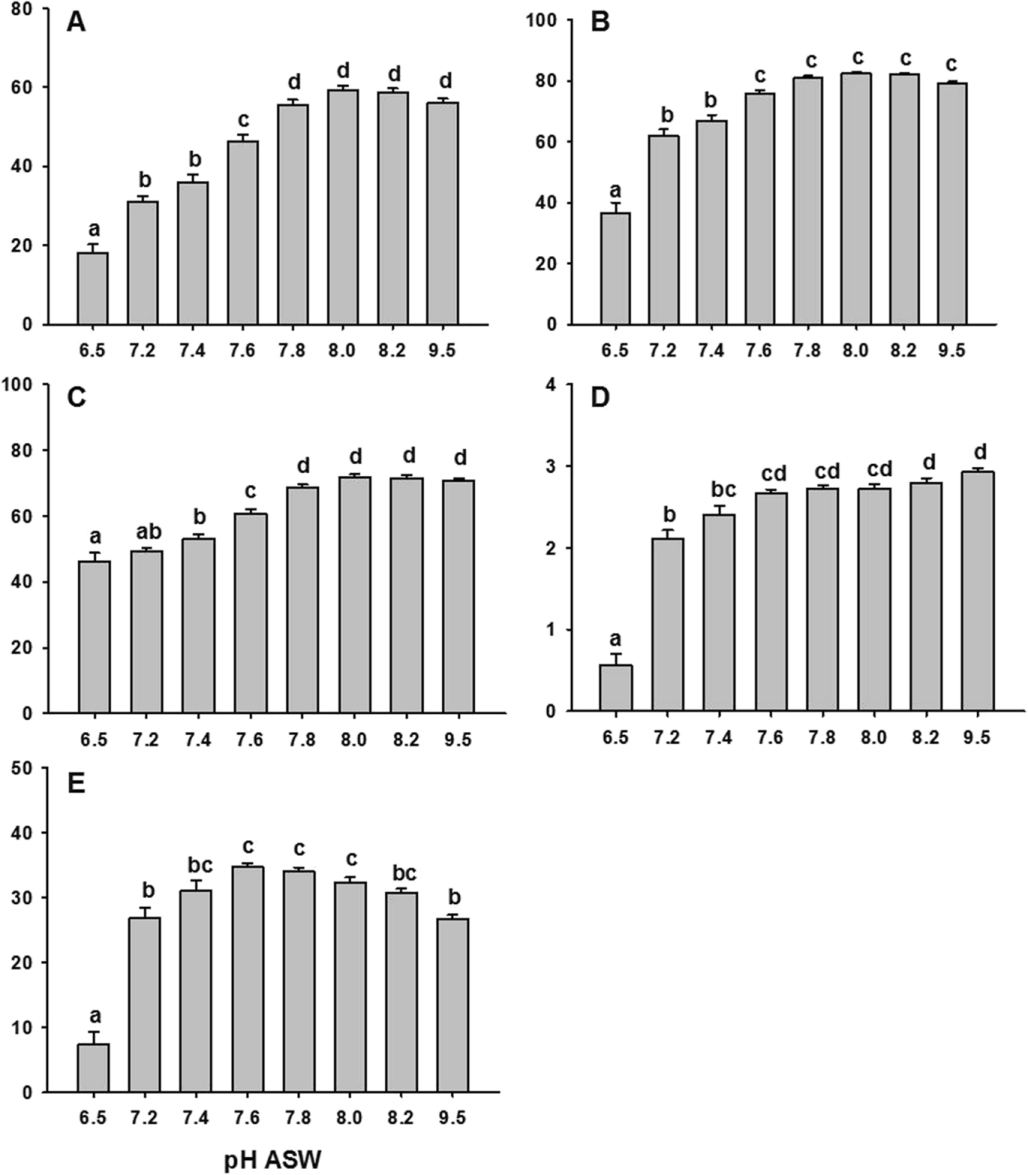
Effect of artificial seawater pH in the linearity index (LIN), straightness (STR, %), oscillation index (WOB), lateral movement of the sperm head (ALH), and cross beating frequency (BCF). Values are represented as means ± standard error. *n* = 23. Different letters indicate significant differences between means (*p* < 0.05)

Regarding other parameters, a progressive increase in LIN, STR, and WOB values could be observed (Fig. 2) when the pH increased from 6.5 to 7.8, without a decrease at the high pH (9.5). The ALH and the BCF were deeply reduced at the low pH (6.5) in relation to the other pH values. The maximum BFC was observed at pH 7.6–8.2, being reduced both at lower and higher pHs.

**Fig. 3:**
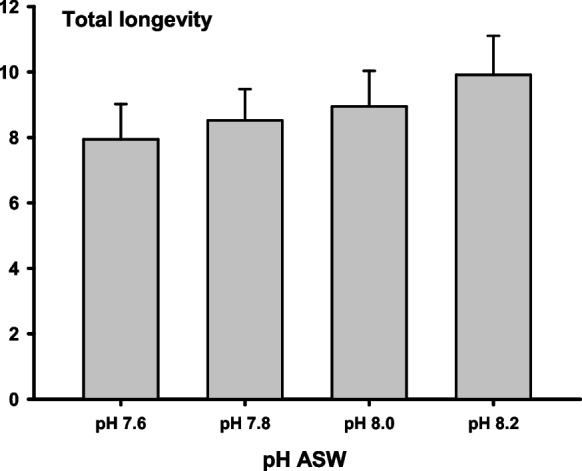
Total longevity of the eel sperm motility (min) activated at different pH of the artificial seawater. Values are represented as means ± standard error. *n* = 10

Figure 3 shows the longevity of sperm motility at different pHs. A gradual but not significant increase in longevity was observed when the ASW pH increased from 7.6 to 8.2.

### Experiment 2. The combined effect of seawater pH and diluent pH on sperm motility

**Fig. 4:**
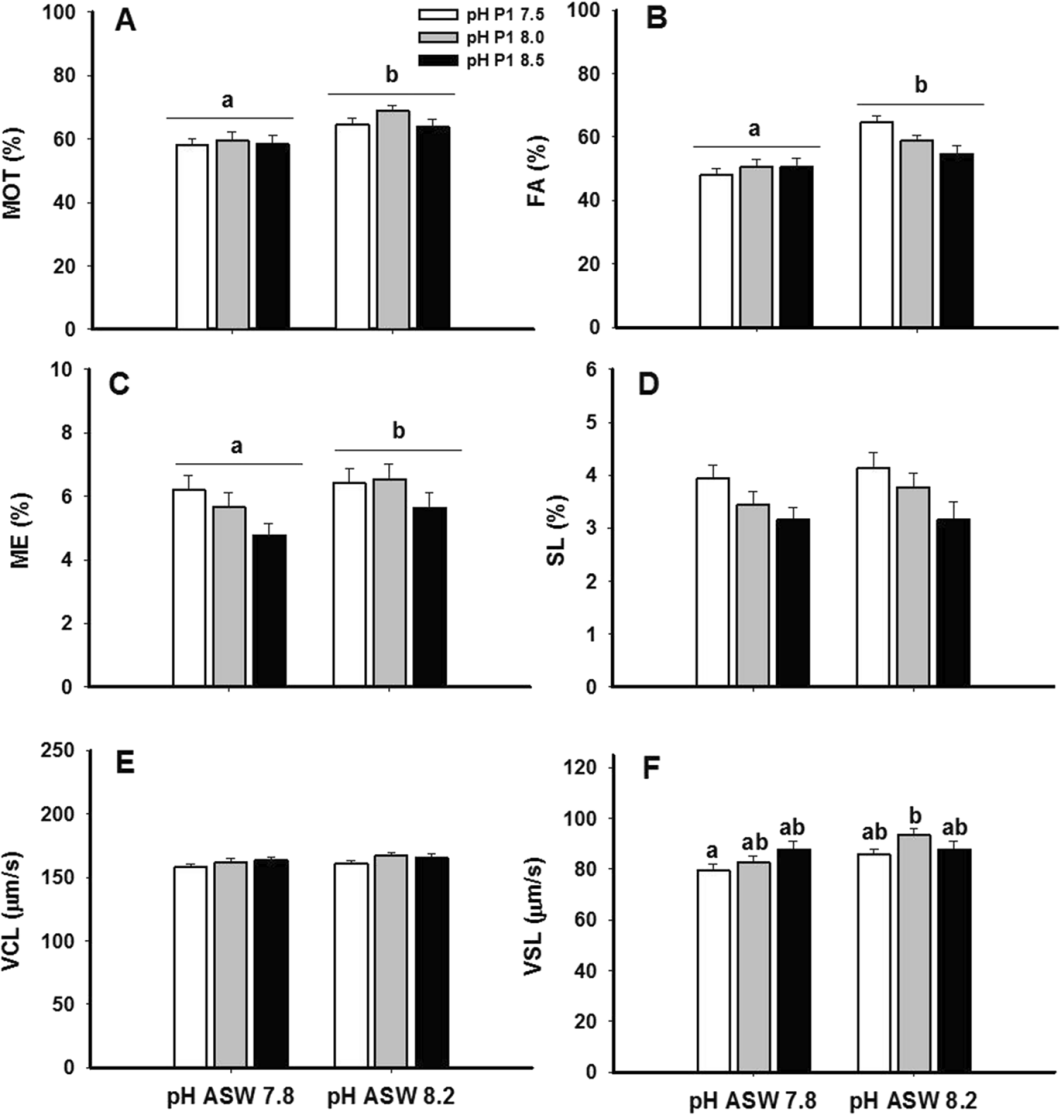
The combined effect of the extender pH with the ASW pH in the total motility (MOT), the percentage of fast (FA), medium (ME) and slow (SL) spermatozoa, the curvilinear velocity (VCL), and the straight line velocity (VSL). Values are represented as means ± standard error. *n* = 16. Different letters indicate significant differences between means (*p* < 0.05)

The aim of this trial was to check the potential interactions between the pH of the artificial seminal plasma and the ASW pH on the eel sperm motility. In Fig. 4, it could be observed that in general, there were no statistical differences in the sperm motility and kinetic parameters in relation to the artificial seminal plasma pH, but seawater pH had a significant effect, showing higher values of total motility (MOT), FA, and ME with a pH of 8.2 than with a pH of 7.8.

The straightline velocity (VSL, Fig. 4F) was affected by the extender pH, with higher velocity in samples diluted at pH 8.0 and activated with ASW at pH 8.2, although the differences were significant only with the samples diluted in extender at pH 7.5 and activated with ASW at 7.8.

**Fig. 5:**
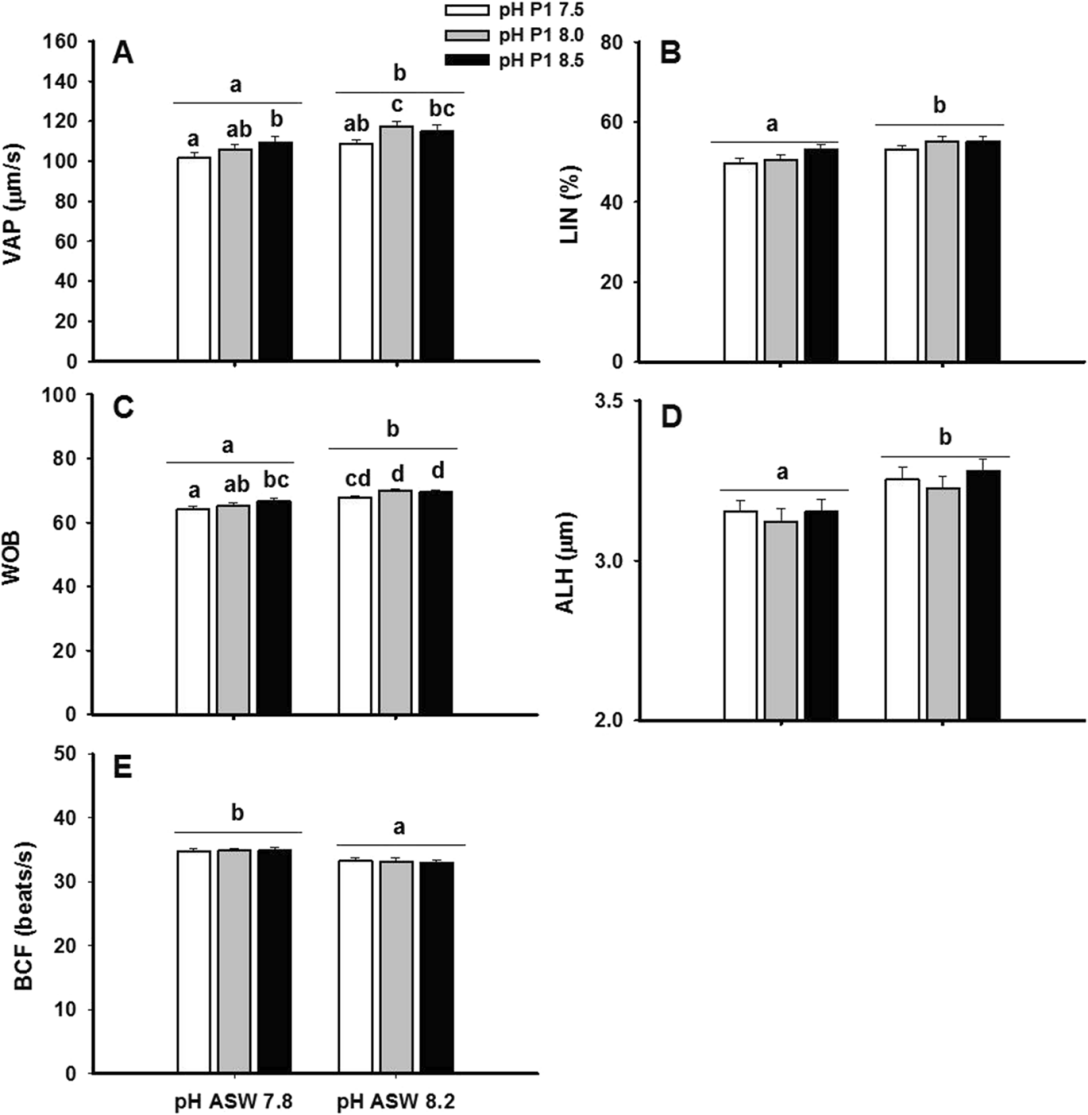
The combined effect of the diluent pH with the ASW pH in the average path velocity (VAP), the linearity index (LIN), oscillation index (WOB), lateral movement of the sperm head (ALH), and cross beating frequency (BCF). Values are represented as means ± standard error. *n* = 16. Different letters indicate significant differences between means (*p* < 0.05)

Figure 5 shows the effects of the combined extender pH and the ASW pH on other kinetic parameters. There was a significant interaction between the extender pH and the ASW pH in VAP and WOB, which lowest values were observed in the samples diluted with the extender at pH 7.5 and then activated with ASW at pH 7.8. The LIN, ALH, and BCF values were only affected by the ASW pH, with lower values of LIN and ALH at pH 7.8 than at 8.2, but higher values of BCF at ASW at pH 7.8 than at 8.2.

### Experiment 3. Effect of seawater temperature on sperm motility and kinetic parameters

**Fig. 6:**
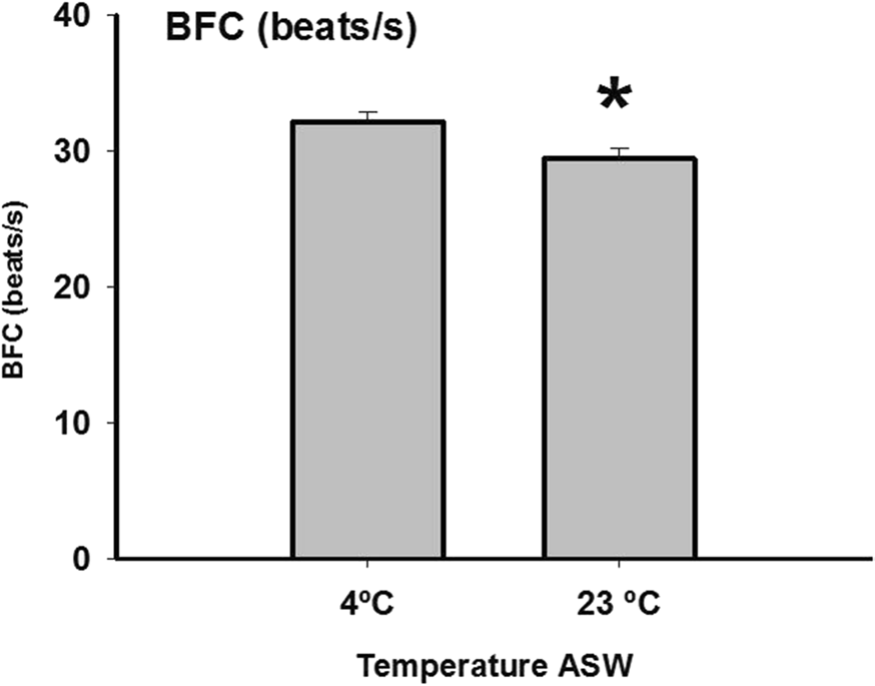
Effect of seawater temperature (ASW) in the beat cross frequency of eel sperm. Values are represented as means ± standard error. *n* = 10. Asterisks indicate significant differences between means (*p* < 0.05)

In this experiment, sperm activation was performed at two temperatures: 4 °C (control) and 23 °C. It should be noted that both the samples and the ASW were maintained at the same temperature. No significant differences were found in MOT, MP, FA, VCL, VSL, VAP, ME, SL, LIN, STR, WOB, and ALH (supplementary data). However, there was an effect of the temperature on the BCF, which was lower (*p* < 0.01) in the samples activated at a high temperature, 23 °C (Fig. 6).

### Experiment 4. Effect of seawater temperature on sperm longevity

This experiment checked the effect of different ASW temperatures (4 and 23 °C) on sperm longevity. Although decreasing trend longevity was observed with the highest temperature, no significant differences were found between the two tested temperatures (data not shown).

### Experiment 5. Combined effect of pH and seawater temperature on sperm motility

**Fig. 7:**
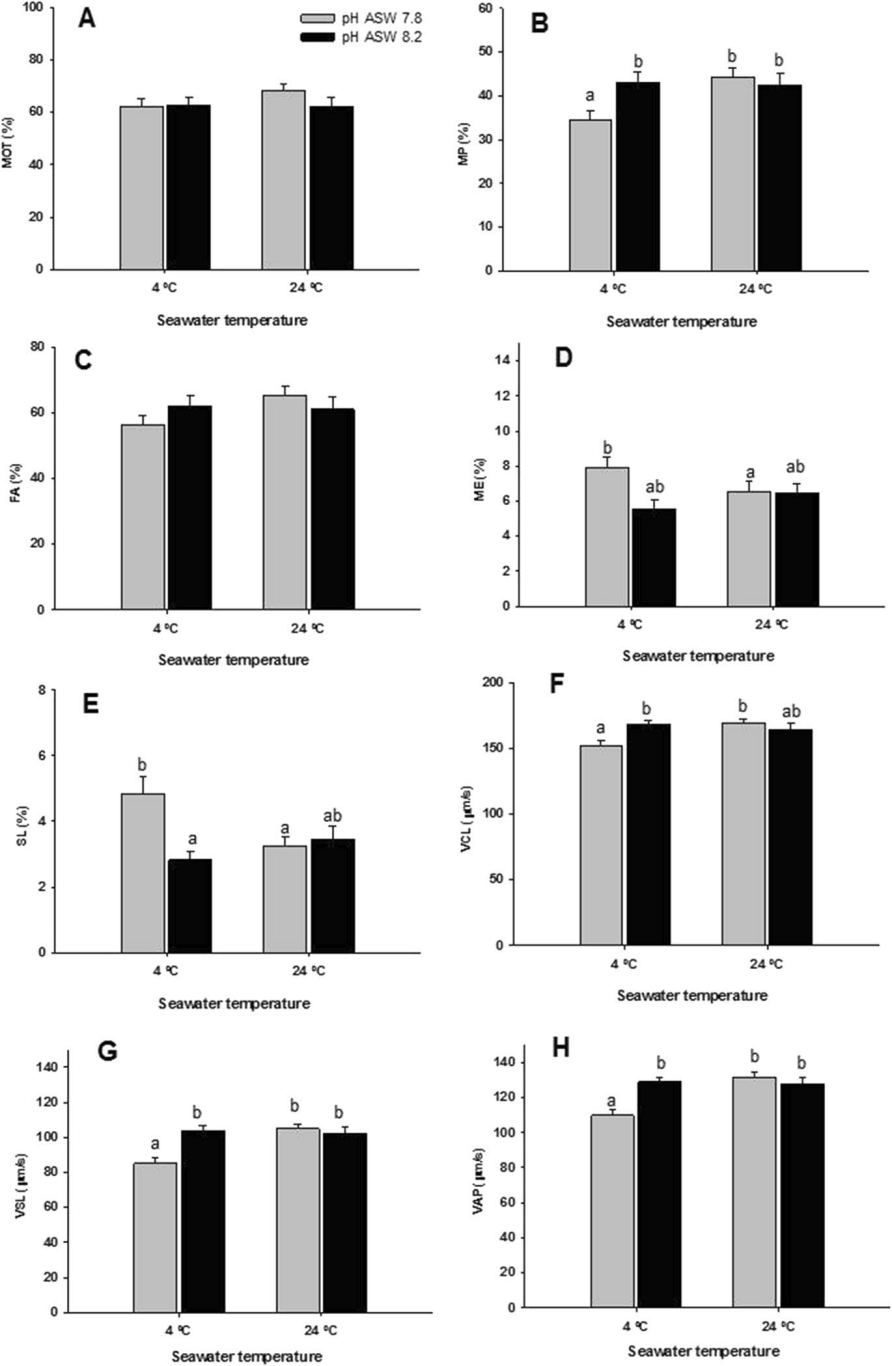
Effect of temperature and pH of seawater (ASW) on the total motility (MOT), progressive motility (MP), the percentage of fast (FA), medium (ME) and slow (SL) spermatozoa, the curvilinear velocity (VCL), and the straight line velocity (VSL). Values are represented as means ± standard error. *n* = 12. Different letters indicate significant differences between the means

With the aim of studying the effect of the interaction between seawater temperature and pH on sperm motility, combinations of two temperatures: 4 and 24 °C, and two pH: 7.8 and 8.2, were tested. Total motility was not affected by temperature or pH (Fig. 7A). However, as can be observed in Fig. 7 B, D, E, and F, there were significant interactions between temperature and pH in several kinetic parameters, such as MP, VCL, VSL, and VAP, in which the lowest values were observed in the samples activated at 4 °C and pH 7.8. Accordingly, the highest values for ME and SL were also observed in the samples activated at 4 °C and pH 7.8 (Fig. 7D, E).

**Fig. 8:**
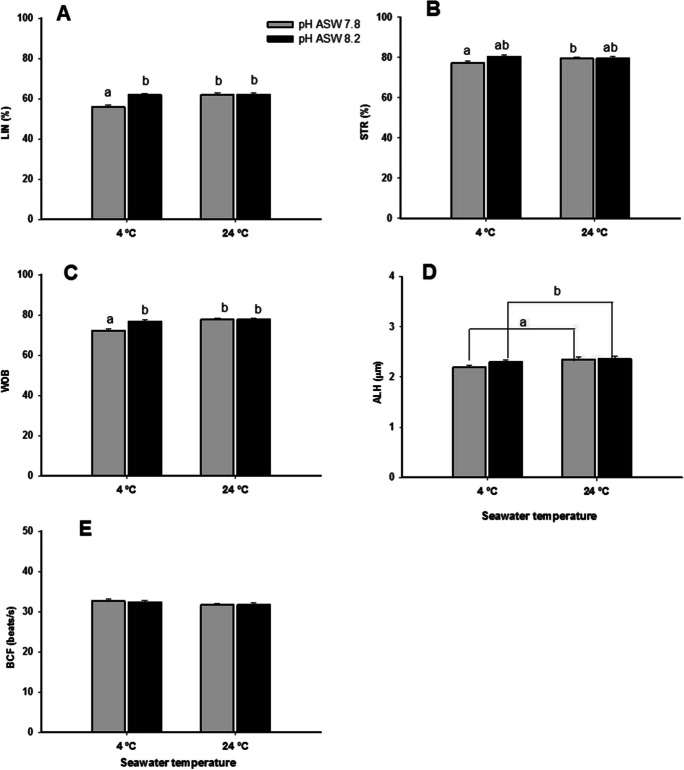
Effect of seawater temperature and pH (ASW) on the linearity index (LIN), oscillation index (WOB), lateral movement of the sperm head (ALH), and cross beating frequency (BCF). Values are represented as means ± standard error. *n* = 12. Different letters indicate significant differences between the means

As for MP, other kinetic parameters (LIN, STR, and WOB) showed their lowest values in the samples activated at 4 °C and pH 7.8 (*p* ≤ 0.05, Fig. 8). Regarding the lateral displacement of the head (ALH), it was influenced by the seawater pH, with lower values for ASW at pH 7.8 than at 8.2 (Fig. 8D).

## Discussion

Due to climate change, sea acidification may mean a decrease of pH from current levels (pH 8.2) to values of 7.7–7.8 in the coming years (Hartin et al. 2016). As a consequence, marine organisms may suffer serious effects, such as a decrease in sperm motility, which is considered one of the main indicators of seminal quality (Gallego and Asturiano 2018, 2019). Eel sperm motility, as well as the sperm motility of many other marine species, could be affected by such a change only in the case that pH decrease below 7.8, as motility was reduced at more acidic pHs (Pérez et al. 2020).

The results in this study indicate that the pH range of 7.8–8.2 in seawater is optimal for maintaining a good European eel sperm movement capacity, producing high values of motility and velocities (MOT, MP, FA, VCL, VSL, and VAP), although some evidence (experiment 2) indicates that a pH of 7.8 induces small but significantly lower motility than a pH of 8.2. The present results agree with the linear regression found by Pérez et al. (2020) between seawater pH and sperm motility, where the lowest values were found at the low pH and the highest at the high pH tested, 8.2. According to the results obtained, it seems that European eel spermatozoa can tolerate the acidification of seawater at least until a pH of 7.8 without any modification of its kinetic parameters.

Moreover, similar results were found for the Japanese eel (Tanaka et al. 2004), where acidification of the activating medium to levels of 7.2 decreased sperm motility to a great extent. Furthermore, studies carried out with salmonids (Ciereszko et al. 2010) and sea trout (*Salmo trutta m. trutta* L.) (Dziewulska and Domagała 2013) also suggest that the low pH of the activating medium affects sperm motility. The reason for the decrease in sperm motility at low seawater pHs could be that dynein, the motor of the flagellum, cannot act at low pH, as was observed in the steelhead trout (*Oncorhynchus mykiss*) (Woolsey and Ingermann 2003). Another possibility is that a lower extracellular pH in the seawater, perhaps inhibits an ion channel such as the potassium ones, the proton pump, or the ion exchangers necessary for the motility initiation.

For instance, a potassium channel involved in sperm motility, the CNKG, has been described in zebrafish, and it is activated by alkalinization (Fechner et al. 2015). The change of membrane potential is another factor that influences fish sperm activation, and it was demonstrated that resting membrane potential was dependent on pH, besides K^+^ and Na^+^, in rainbow trout (*Salmo gairdneri*) (Catti et al. 1990). In mammals, a proton pump seems to be involved in sperm motility (Escoffier et al. 2020). Thus, a low seawater pH could inhibit the ionic exchanges and the subsequent membrane potential change necessary for the motility activation.

Also, a decrease in the pH of seawater can limit the metabolic activity of gametes and reduce the mitochondrial membrane potential of spermatozoa, hindering flagellum motility (Catti et al. 1990; Alavi and Cosson 2005; Ciereszko et al. 2010; Dziewulska and Domagała 2013; Castro-Arnau et al. 2022). The alkalinization of the seminal plasma is a necessary process for the activation of sperm movement in most fish (Alavi and Cosson 2005). Our results showed no difference in the effect of the pH of the extender (artificial seminal plasma) on sperm kinetics, but seawater pH significantly affected motility, showing better kinetic parameters for pH 8.2 than 7.8. In the case of the European eel, sperm motility could be affected by such a change, as motility was reduced at acidic pHs, similar to our previous results (Gallego et al. 2014; Vílchez 2017; Pérez et al. 2020), as well as what has been observed with the sperm motility of the Japanese eel (Tanaka et al. 2004).

Previous research has reported that internal pH influences spermatozoan maturation and the motility of ejaculated spermatozoa in several fish species (Catti et al. 1990; Gallis et al 1991; Ciereszko et al. 2010; Dziewulska and Domagała 2013; Kutluyer 2018; Castro-Arnau et al. 2022). Studies undertaken by Pickering and Pottinger (1987), demonstrated that sperm exposure to a reduced pH for brown trout (*Salmo trutta*) shows elevated ammonia levels in combination with significantly increased plasma cortisol levels, which can lead to poor sperm quality. Pérez et al. (2020) observed in European eel that the change in the intracellular pH of sperm cells at activation is linearly dependent on the pH of the diluent medium; this can favor the progressive movements and rectilinear velocity of the spermatozoa when they are activated with seawater with pH 8.2 as found in our present study.

The results of several studies suggest that the temperature of the medium used in motility activation is directly involved in the sperm motility characteristics of many fish species with either internal or external fertilization (Dadras et al. 2017; Fenkes et al. 2017; Merino et al. 2023), such as carp (*Cyprinus carpio*) (Cejko et al. 2016) or brown trout (Fenkes et al. 2017).

In the present study, the effect of the seawater temperature on sperm motility and kinetic parameters was evaluated, and according to the results obtained, the different temperatures examined (4 and 24 °C) did not significantly affect the motility of the European eel sperm. In fact, the only kinetic parameter that presented significant differences was BCF, which was lower at the high temperature, indicating a negative effect. The effect of seawater temperature on sperm longevity was not observed. Similar results were found for gilthead seabream (*Sparus aurata*) spermatozoa, where it was demonstrated that the initial motility parameters were not affected by a temperature range of 4–22 °C (Lahnsteiner and Caberlotto 2012).

When we combined the effect of pH and seawater temperature on sperm motility, we observed a trend in which the lowest pH (7.8) combined with the lowest seawater temperature (4 °C) caused a reduction of the European eel sperm motility. Higher temperatures seem to favor the sperm quality of the species when combined with a more alkaline pH of the activating medium (pH 8.2). Although seawater pH can influence the initiation and duration of sperm motility, according to our results, the pH in the range 7.8–8.2 does not appear to affect the duration of movement of the European eel spermatozoa, which is possibly more resistant than other species to pH variation.

Studies on different fish species, mostly from temperate or cold waters, indicate there is an inverse relationship between environmental temperature and the duration of sperm motility, which could be the result of expenditure of limited energy stores available for motility (Alavi and Cosson 2005; Dadras et al. 2017). For Nile tilapia (*Oreochromis niloticus*) spermatozoa, environmental temperature induces an increase of velocity without a decrease of the percentage motility at an extremely wide range of temperatures (5–50 °C). Flagellar beat frequency would be associated with an increasing ATP utilization and generation rate with increasing temperatures (Dzyuba et al. 2019).

Our results indicate that the pH 8.2 and a temperature of 4 °C is adequate to be used as standard in the European eel sperm activating medium. Temperature does not appear to affect the duration of sperm movement, but the combination of low temperature and low pH induced lower sperm motility. The present results indicate that in the context of climate change, the European eel sperm could experience pHs lower than the standard (up to 7.8) and higher temperatures than in the spawning area. That is, it can move perfectly at 23–24 °C, while the supposed temperature in the spawning area is 20 °C (van Ginneken and Maes 2005). This work suggest that eel sperm motility and kinetics will not be affected by the expected changes in pH or temperature due to the climate change.

## Data Availability

All data will be accessible by contacting with the corresponding author.
